# Relevance between patients’ clinical factors and 4D-CT lung ventilation heterogeneity for evaluating the adoption of functional avoidance thoracic radiotherapy with a large patient database

**DOI:** 10.3389/fonc.2026.1955295

**Published:** 2026-10-07

**Authors:** Guozhong Deng, Yongquan Ying, Xiaomei Wang, Xingni Tang, Shi Su, Changhui Yu, Pingjun Gu, Haihua Yang

**Affiliations:** 1 Key Laboratory of Radiation Oncology of Taizhou, Radiation Oncology Institute of Enze Medical Health Academy, Department of Radiation Oncology, Taizhou Hospital Affiliated to Wenzhou Medical University, Linhai, Zhejiang, China; 2 Department of Thoracic Surgery, Taizhou Hospital Affiliated to Wenzhou Medical University, Linhai, Zhejiang, China

**Keywords:** 4D-CT, lung function, radiation pneumonitis, radiotherapy, ventilation

## Abstract

**Objective:**

Four-dimensional computed tomography (4D-CT)-guided functional-avoidance radiotherapy reduces radiation pneumonitis, but the lack of patient selection criteria limits its adoption. This study aimed to identify clinical predictors of lung ventilation heterogeneity and determine which patients derive the greatest dosimetric benefit from functional-avoidance planning.

**Methods:**

We retrospectively analyzed 274 lung cancer patients. 4D-CT ventilation imaging was generated via deformable registration and a density-change algorithm, and the coefficient of variation (CoV) across six lung zones quantified heterogeneity. Clinical factors were assessed using Mann-Whitney U, Spearman, and multivariable logistic regression for three ventilation outcomes. Thirty-two patients were selected from CoV quartiles and re-planned with functional-avoidance plans; dosimetric parameters were compared using the Wilcoxon signed-rank test, with False Discovery Rate (FDR) correction applied to address multiple comparisons.

**Results:**

Univariate analysis showed chronic obstructive pulmonary disease (COPD) and airway obstruction reduced bilateral mean ventilation, while central tumors impaired ipsilateral ventilation. Higher CoV was observed in stage III-IV versus I-II, central versus peripheral tumors, and patients with airway obstruction. Multivariable analysis identified airway obstruction as an independent predictor of elevated CoV (adjusted OR = 2.914, 95% CI: 1.315–6.454, P<0.05). Central location and advanced stage showed borderline significance, while COPD trended toward lower CoV. Dosimetrically, functional-avoidance plans reduced functional lung metrics in a heterogeneity-dependent manner. After FDR correction, key metrics (fMLD, fV10, fV20, fV30) remained significant in Group 1 (highest CoV; Q = 0.040–0.049), while significance diminished in lower CoV groups. Leave-one-out (LOO) sensitivity analysis confirmed fMLD and fV20 as robust endpoints. Furthermore, beam arrangement modifications were most frequent in Group 1 (5/8 patients) and absent in Group 4 (0/8), reflecting clinical optimization needs rather than confounding.

**Conclusions:**

Ventilation heterogeneity is driven by tumor burden and airway patency. Patients with advanced stage, central tumors, or airway obstruction exhibit the strongest heterogeneity and may represent appropriate candidates for functional-avoidance radiotherapy in future prospective trials. However, as this replanning study lacked clinical follow-up for radiation pneumonitis, these findings reflect dosimetric rather than confirmed clinical patient-level benefit. Notably, CoV quartiles were derived from internal median splits within this cohort and require external validation. This study provides a dosimetric basis for hypothesis-generating patient selection.

## 1 Introduction

Radiation pneumonitis imposes a critical constraint on the maximal achievable prescription dose in thoracic radiotherapy: for patients with large tumor burdens requiring higher doses for adequate tumor control, the need to maintain lung doses within safe thresholds to avoid radiation pneumonitis often precludes delivery of the required prescription dose, ultimately compromising therapeutic efficacy. Consequently, minimizing the incidence of radiation pneumonitis while ensuring adequate target coverage has emerged as a pivotal research priority in thoracic radiotherapy (1–3). Functional-avoidance radiotherapy can reduce the radiation dose to the highly functional lung, thereby achieving the goal of decreasing the incidence of radiation pneumonitis (4, 5). In recent years, the proliferation of four-dimensional computed tomography (4D-CT) lung ventilation function imaging technology has revolutionized the landscape (6–9). Being a routine and cost-effective diagnostic tool for lung cancer patients, it has replaced single-photon emission computed tomography (SPECT) and positron emission tomography/computed tomography (PET/CT) as the go-to modality for assessing lung function and has emerged as a pivotal imaging tool in lung functional-avoidance radiotherapy (10).

The 4D-CT lung ventilation imaging has been widely demonstrated to effectively reflect the regional lung ventilation status of patients (11–15). Therefore, it is currently widely implemented in functional-avoidance radiotherapy for lung cancer (16). The current applications of 4D-CT ventilation imaging in lung cancer radiotherapy include the assessment of lung ventilation distribution and its changes after radiotherapy (17–19), precise prediction of radiation pneumonitis with 4D-CT functional dose metrics (20–22) and the research of dosimetric advantages in functional-avoidance radiotherapy (23–25). There are also multiple ongoing clinical trials aiming at evaluating the effectiveness of 4D-CT functional-avoidance radiotherapy in reducing radiation pneumonitis in patients (16), with some of these clinical studies already yielding exciting outcomes (26–28).

It is worth noting that while most of the current studies of functional-avoidance radiotherapy have consistently demonstrated its ability to reduce lung function loss and the incidence of radiation pneumonitis in patients (4, 5, 23–29), a small number of studies show that it has failed to effectively reduce the dose to the highly functional lung and the incidence of radiation pneumonitis (5, 30). This latter observation may stem from the weak heterogeneity of lung ventilation function among patients in those studies, leaving little room for sparing. Consequently, in recent clinical trials of 4D-CT functional-avoidance radiotherapy, researchers have imposed certain requirements on the heterogeneity of patients’ ventilation distribution when selecting candidates for this treatment approach (27). Vinogradskiy et al. reported a grade ≥2 radiation pneumonitis rate of 14.9% with functional-avoidance planning (27), supporting the clinical relevance of ventilation-based patient selection. Subsequent quality-of-life analysis of the same cohort further demonstrated that only 26.8% and 29.3% of patients experienced clinically meaningful declines in FACT-TOI and FACT-LCS at 12 months post-treatment, respectively (31), reinforcing the patient-centered benefit of this approach.​ These findings underscore that the heterogeneity of lung ventilation has become an essential criterion for determining the necessity of functional-avoidance radiotherapy.

However, the factors that drive ventilation heterogeneity and the extent to which heterogeneity translates into dosimetric benefit remain incompletely understood.​ A prior study examined 4D-CT regional ventilation distribution in stage I and III lung cancer patients (17), focusing primarily on the correlation of ventilation defects with chronic obstructive pulmonary disease (COPD) and airway obstruction. This work did not, however, evaluate the impact of a broader range of clinical factors on ventilation heterogeneity, nor did it explore the dosimetric benefits of functional-avoidance planning across different degrees of heterogeneity.​ Additionally, emerging evidence suggests that central tumors may particularly benefit from functional lung avoidance Stereotactic Body Radiation Therapy (SBRT) (32), further motivating the need to identify clinical predictors of ventilation heterogeneity.

To address these gaps, this study aims to broaden the scope by investigating the impacts of a wide variety of clinical factors on the regional distribution and heterogeneity of lung ventilation for lung cancer patients. Specifically, we seek to unravel the correlation between the heterogeneity of lung ventilation and the dosimetric benefits for patients undergoing functional-avoidance radiotherapy, thereby providing a dosimetric basis for hypothesis-generating patient selection in future prospective trials of functional-avoidance radiotherapy in lung cancer patients.

## 2 Materials and methods

### 2.1 Patient data

This retrospective study was conducted in accordance with the Declaration of Helsinki and was approved by the Medical Ethics Committee of Taizhou Hospital Affiliated to Wenzhou Medical University (Approval No. K20240822). The requirement for informed consent was waived due to the retrospective observational nature of this study. A total of 274 cases of lung cancer of stage I to IV, who underwent radiotherapy in the Radiotherapy Department of Taizhou Hospital Affiliated to Wenzhou Medical University from March 2018 to April 2024, are included in this study. Metastatic lung cancer is excluded from the case selection to ensure that all enrolled patients had primary lung cancer and had undergone 4D-CT scans prior to radiotherapy. Furthermore, rigorous manual screening of the patients’ 4D-CT images is conducted to exclude those with significant volume averaging artifacts. Patients with stage III-IV lung cancer undergo conventional External Beam Radiation Therapy (EBRT), while some patients with stage I-II lung cancer receive SBRT, and the remaining stage I-II cases also undergo EBRT. The planning gross tumor volume (PGTV) and planning target volume (PTV) prescription doses for patients undergoing EBRT range from 54 to 64.8 and 45 to 60 Gy respectively, administered in 25 to 30 fractions, five times a week. For patients receiving SBRT, the administered PGTV dose ranges from 30 to 50 Gy, delivered in 5 to 10 fractions, also five times a week. Note that the PGTV is not given a prescription dose for some patients undergoing EBRT.

### 2.2 4D-CT Ventilation calculation

Each patient’s pre-radiotherapy 4D-CT images are used to calculate the lung ventilation images. This computation employs a method based on changes in Hounsfield Unit (HU) density (6, 7). The process begins by segmenting the lung structure from the end-inspiration and end-expiration 4D-CT images. Deformable image registration was performed with Plastimatch implemented in the 3D-Slicer platform. A cubic B-spline registration algorithm with second-derivative regularization was used to estimate the deformation vector field mapping the end-expiration image to the end-inspiration image. The cost function combined a mean-squared-error intensity term and a smoothness regularizer, optimized with the limited-memory BFGS with bounds algorithm in a three-stage hierarchical scheme; the finest B-spline control-point grid spacing was 10 × 10 × 20 mm³ and the regularization weight was λ = 0.01, selected by qualitative assessment of deformation quality. This parameter configuration followed the Plastimatch B-spline setup reported by Kipritidis et al. (13) and has been used in our previous 4D-CT ventilation study (33). In the validation cohort of Kipritidis et al., landmark displacement error for this type of registration was 1.8 ± 1.5 mm (13); no independent ground-truth deformation was recomputed for the present cohort, so registration accuracy here depends on image quality, respiratory motion amplitude, phase sorting, and parameter settings. Both lung segmentation and the resulting deformation fields were manually inspected to exclude foldings, discontinuities, and obvious phase mismatches before ventilation computation. Once the registration is completed, the second step of ventilation calculation commences. This involves applying a density-change-based algorithm to compute the ventilation using HU values of corresponding voxel points between the registered end-inspiration image and the original end-expiration image, respectively. The mathematical expression for this calculation is shown in Equation 1 (6, 7):

(1) $$\frac{V_{in}-V_{ex}}{V_{ex}}=1000\frac{HU_{in}-HU_{ex}}{HU_{ex}\left(1000-HU_{in}\right)}$$

In the aforementioned equation, *v_in_* and *v_ex_* represent the voxel volumes at end-inspiration and end-expiration, respectively, while *HU_in_* and *HU_ex_* denote the HU values of the corresponding voxels of the end-inspiration and end-expiration images. Typically, water has an HU value of 0, air has an HU value of -1000, and the HU values of lung tissue usually range between -900 and -200. The logic behind this calculation is that the HU value of each lung voxel changes during respiration as the air content in the lungs varies. By analyzing these changes in HU values, we can derive the volume changes of lung voxels, thereby obtaining the distribution of lung ventilation. It should be noted that the specific parameter configuration of the DIR algorithm (e.g., regularization weight λ, grid spacing) can influence the estimated deformation field and thus the calculated CoV, which is further discussed in the Limitations section.

### 2.3 Ventilation-based functional metrics

To investigate the impacts of clinical factors on patients’ regional lung function, we have derived two ventilation-based functional metrics from their 4D-CT lung ventilation images. The first metric is the mean ventilation for the ipsilateral and contralateral lungs of patients, which is used to assess the overall lung ventilation status of patients under different clinical factors. The second metric involves dividing each patient’s total lungs into six regions vertically (three segments per lung, from superior to inferior), and calculating the coefficient of variation (CoV) for the mean ventilation in each of these six regions. This segmentation approach is standard in nuclear medicine imaging (34) and has also gained widespread adoptions in 4D-CT ventilation studies for assessing patients’ regional ventilation patterns (17, 35). This approach enables us to evaluate the impacts of various clinical factors on the heterogeneity of patients’ lung ventilation. To calculate the CoV, we first determine the mean ventilation value for each of the six regions, and then compute the standard deviation (SD) and mean of these six values. The CoV is the ratio of the SD to the mean.

### 2.4 Treatment planning and dose metrics

This study aims to investigate the correlation between patients’ lung ventilation heterogeneity and the dosimetric advantages of functional-avoidance plans over anatomic plans. Consequently, it necessitates the re-planning with functional-avoidance strategies from patients’ anatomic treatment plans. Given that some Stage I-II patients undergo SBRT, which significantly differs from EBRT in terms of dose constraints and other aspects, this work exclusively focuses on the dosimetric differences between functional-avoidance plans and anatomic plans for EBRT. A cohort of 211 patients undergoing EBRT is analyzed, and their CoV of lung ventilation are ranked from highest to lowest, reflecting a gradient from stronger to weaker lung ventilation heterogeneity. Subsequently, utilizing the 25th percentile as a cut-off, we stratify these patients into four groups (designated as Groups 1, 2, 3, and 4, with ventilation heterogeneity from stronger to weaker). From each CoV quartile, eight patients were randomly selected using computer-generated random numbers (fixed seed = 12345), resulting in a total of 32 patients for whom functional-avoidance plans are created.

Both the functional-avoidance plan and the patient’s treatment plan (anatomic plan) utilize intensity-modulated radiation therapy (IMRT) methodology and adhere to identical dose requirements for target volumes and dose constraints for organs at risk (OARs). The dose requirements and constraints are listed as follows: prescription dose covers over 90% of the target volume for PGTV and PTV; V5< 60%, V20< 25% and mean lung dose (MLD)< 20 Gy for lung; V30< 40% and V40< 30% for heart; maximum dose (Dmax) ≤ 45 Gy for spinal cord planning organ at risk volume (PRV); mean dose (Dmean)< 35 Gy and V40< 40% for esophagus; V35< 50% for liver. The Pinnacle planning system (Pinnacle³ version 16.2, Philips Radiation Oncology Systems, Fitchburg, WI, USA) is employed for the creations of these radiotherapy plans. Both the functional-avoidance plan and the original treatment plan are crafted by experienced planners on free-breathing or 4D-CT-averaged images for all the patients. Given the absence of a universally standardized definition for the high-ventilation region, we defined the functional lung as the top 20% of the lung ventilation volume for avoidance planning. This threshold was selected as it falls within the most commonly adopted range (10%–30%) in the field of functional-avoidance radiotherapy (20–28). Prior literature has established that, across a broad range of thresholds, the choice of ventilation threshold does not drastically alter predictive outcomes (21). Given this demonstrated stability, we selected the top 20% as a representative cutoff for functional lung definition in this study. Nevertheless, a systematic sensitivity analysis using alternative thresholds was beyond the scope of the current dataset and remains a limitation of this work. The resulting top 20% ventilation region was then converted into a structure for protection during dose optimization. Planners were permitted to modify beam angles or the number of fields if deemed necessary to achieve meaningful functional avoidance, based on the relative position of the target volumes and the functional lung. Although planners could discern patients with stronger or weaker ventilation heterogeneity by observing the distribution of high-ventilation regions, they were not informed of the specific CoV group assignment when creating the functional-avoidance plans, minimizing human bias.

The comparative evaluations of the dose metrics between functional-avoidance plans and anatomic plans include dose metrics of target volumes and OARs. The dose metrics of the target volumes encompass the homogeneity index (HI) (36) and conformity index (CI) (37) of the PTV. The dose metrics of the OARs include the heart’s V30, V40, and mean dose, the maximum dose to the spinal cord PRV, the mean dose to the esophagus, as well as two types of lung dose metrics. Lung dose metrics consist of conventional entire-lung-based dose metrics and functional lung dose metrics. The conventional lung dose metrics include MLD, V5 (percentage of total lung volume receiving dose more than 5 Gy), V10, V20, and V30. The functional lung dose metrics are the dose metrics based on the functional lung, like fMLD, fV5, fV10, fV20, and fV30 (representing MLD, V5, V10, V20, and V30 within the functional lung).

### 2.5 Data analysis and statistical methods

The initial phase of this research work is to analyze the influences of various clinical factors on regional lung ventilation and its homogeneity. We first performed univariate analysis: we utilize the Mann-Whitney U test to examine the associations between binary clinical factors and ventilation-based metrics, while adopting Spearman’s correlation analysis to delve into the influences of continuous clinical factors on the ventilation-based metrics. Subsequently, multivariable logistic regression was performed to identify independent predictors. Variance inflation factor (VIF) was calculated using a linear regression model to assess multicollinearity among all covariates, with VIF< 5 considered indicative of no significant multicollinearity. For the ipsilateral and contralateral lung ventilation analyses, the dependent variable was low mean ventilation (< median, coded as 1) versus good ventilation (≥ median, coded as 0). For the CoV analysis, the dependent variable was high CoV (≥ median, coded as 1) versus low CoV (< median, coded as 0). The following variables were entered into the multivariable models: sex (female as reference), stage (I-II as reference), tumor location (peripheral as reference), COPD status (no as reference), smoking history (no as reference), airway obstruction (no as reference), age (per 10 years), and GTV volume (per 10 cm³). Airway obstruction was assessed on pretreatment diagnostic CT or planning 4D-CT images by evaluating direct signs (luminal narrowing, abrupt cutoff, intraluminal soft-tissue density, extrinsic compression) and indirect signs (distal atelectasis, obstructive pneumonia, mucus plugging) in the proximal tracheobronchial tree at the tumor site. Initial assessment was performed by a radiation oncologist, and all cases were subsequently reviewed and confirmed by a radiologist, with discrepancies resolved through joint review. The final binary classification (YES/NO) was recorded accordingly.​ Sensitivity analyses were performed by dichotomizing GTV and age at their medians to confirm the robustness of the continuous-variable results. Lung volume was excluded from the final multivariable models due to lack of independent predictive values (adjusted OR ≈ 1.000 per 1 cm³). Moving forward, the second phase focuses on elucidating the dosimetric benefits of functional-avoidance plans over anatomic plans in patients exhibiting varying degrees of lung ventilation heterogeneity. We adopt the Wilcoxon signed-rank test to analyze the statistical differences in dose metrics of target volumes and OARs between the functional-avoidance plans and the anatomic plans. For the 32 re-planned patients, each patient served as their own control (paired analysis). Given the multiple paired comparisons across four subgroups, we performed False Discovery Rate (FDR) correction using the Benjamini–Hochberg (BH) procedure. Recognizing that conventional lung metrics and functional lung metrics represent two distinct families of hypotheses, we applied the BH procedure separately within each family (m=5 for each) to control the FDR within each family while avoiding cross-family penalty.​ Additionally, a Leave-One-Out (LOO) sensitivity analysis was conducted for functional dose metrics that remained significant after FDR correction in Group 1. For each metric, one patient was sequentially removed based on the rank of absolute paired differences, and the Wilcoxon signed-rank test was re-run on the remaining 7 patients to assess robustness.​ The statistical analysis is performed using SPSS 27.0 software (IBM Corp., Armonk, NY, USA), and P< 0.05 was considered statistically significant for raw comparisons, while FDR-adjusted Q< 0.05 was considered significant after correction. All statistical tests are two-sided.

## 3 Results

### 3.1 Correlation of the regional distribution of lung ventilation function and clinical factors

**Table 1** summarizes the primary clinical characteristics of the 274 patients included in the study.​ Among them, 207 (75.6%) were male and 67 (24.4%) were female; 213 (77.7%) had advanced stage (III–IV) and 61 (22.3%) had early stage (I–II); 179 (65.3%) had central tumors and 95 (34.7%) had peripheral tumors; 144 (52.6%) had COPD and 130 (47.4%) did not; 43 (15.7%) had airway obstruction and 231 (84.3%) did not. The mean GTV volume was 60.3 ± 71.16 cm³, mean PTV volume was 241.6 ± 173.2 cm³, and mean total lung volume was 3542.8 ± 930.3 cm³.

**Table 1:** The main clinical characteristics of the patients included in the study.

Characteristic	Category	n (%)
Age	<75	225 (82.1%)
	≥75	49 (17.9%)
Sex	Male	207 (75.6%)
	Female	67 (24.4%)
Stage	Early (I-II)	61 (22.3%)
	Advanced (III-IV)	213 (77.7%)
Location	Central	179 (65.3%)
	Peripheral	95 (34.7%)
Pathology	NSCLC	201 (73.4%)
	SCLC	73 (26.6%)
COPD	YES	144 (52.6%)
	NO	130 (47.4%)
Smoke	YES	164 (59.9%)
	NO	110 (40.1%)
Airway obstruction	YES	43 (15.7%)
	NO	231 (84.3%)
GTV volume (cm^3^)	Mean ± SD	60.3 ± 71.16
PTV volume (cm^3^)	Mean ± SD	241.6 ± 173.2
Lung volume (cm^3^)	Mean ± SD	3542.8 ± 930.3

NSCLC, non-small cell lung cancer; SCLC, small cell lung cancer; COPD, chronic obstructive pulmonary disease; GTV, gross tumor volume; PTV, planning target volume.

The univariate and multivariable analyses of clinical factors associated with ipsilateral and contralateral mean ventilation are presented in **Tables 2** and **3**, respectively.

**Table 2:** The correlation between various clinical factors and the mean ventilation of the ipsilateral lung: univariate and multivariable analysis.

Characteristics	Ventilation, ipsilateral	Univariate *P*	Adjusted OR (95% CI)	Multivariable *P*
Sex		0.896	0.883 (0.462-1.689)	0.707
Male	0.152 ± 0.0433			
Female	0.1479 ± 0.0412			
Stage		0.539	0.972 (0.509-1.853)	0.930
I-II	0.1491 ± 0.0437			
III-IV	0.1516 ± 0.0426			
Location		< 0.05*	2.282 (1.32-3.945)	< 0.05*
Central	0.139 ± 0.0383			
Peripheral	0.1574 ± 0.0437			
COPD		< 0.05*	2.446 (1.386-4.317)	< 0.05*
Yes	0.1415 ± 0.0416			
No	0.1617 ± 0.0417			
Smoke		0.132	0.786 (0.424-1.458)	0.445
Yes	0.1547 ± 0.0434			
No	0.1456 ± 0.0413			
Airway obstruction		< 0.05*	2.58 (1.185-5.618)	< 0.05*
Yes	0.129 ± 0.0402			
No	0.1552 ± 0.042			
Age		0.717 (r = -0.022)	1.072 (0.801-1.424)	0.637
GTV volume		< 0.05* (r = -0.147)	1.116 (0.825-1.495)	0.489
Lung volume		0.423 (r = -0.049)		Excluded**

Data are presented as mean ± SD. Low ipsilateral ventilation was defined as< median and coded as 1; good ventilation (≥ median) was coded as 0. *P< 0.05. **Lung volume was excluded from the final multivariable model (adjusted OR = 1.000 per 1 cm³, 95% CI: 0.996–1.004, P = 0.899). GTV volume is reported per 10 cm³ increase; age is reported per 10-year increase. VIF values for all covariates in the multivariable model ranged from 1.011 to 1.12 (all< 5), indicating no significant multicollinearity. Sensitivity analysis using median-split categories for GTV and age yielded consistent directions of association with the main continuous-variable analysis. Reference groups and statistical methods are described in the Data analysis and statistical methods section.

**Table 3:** The correlation between various clinical factors and the mean ventilation of the contralateral lung: univariate and multivariable analysis.

Characteristics	Ventilation, contralateral	Univariate *P*	Adjusted OR (95% CI)	Multivariable *P*
Sex		0.880	0.986 (0.538-1.809)	0.965
Male	0.1678 ± 0.0478			
Female	0.1673 ± 0.0458			
Stage		0.259	0.863 (0.47-1.586)	0.636
I-II	0.1629 ± 0.0472			
III-IV	0.1691 ± 0.0473			
Location		0.101	1.666 (0.917-3.025)	0.094
Central	0.1601 ± 0.0425			
Peripheral	0.1717 ± 0.0492			
COPD		< 0.05*	2.03 (1.191-3.459)	< 0.05*
Yes	0.1568 ± 0.0446			
No	0.1799 ± 0.0473			
Smoke		0.301	0.76 (0.388-1.488)	0.423
Yes	0.1704 ± 0.0482			
No	0.1637 ± 0.0457			
Airway obstruction		< 0.05*	3.531 (1.606-7.761)	< 0.05*
Yes	0.1437 ± 0.037			
No	0.1722 ± 0.0477			
Age		0.266 (r = -0.068)	1.062 (0.817-1.397)	0.639
GTV volume		0.126 (r = -0.093)	1.072 (0.745-1.553)	0.713
Lung volume		0.429 (r = -0.048)		Excluded**

Data are presented as mean ± SD. Low contralateral ventilation was defined as< median and coded as 1; good ventilation (≥ median) was coded as 0. *P< 0.05. **Lung volume was excluded from the final multivariable model (adjusted OR = 1.000 per 1 cm³, 95% CI: 0.996–1.004, P = 0.907). GTV volume is reported per 10 cm³ increase; age is reported per 10-year increase. VIF values for all covariates in the multivariable model ranged from 1.011 to 1.12 (all< 5), indicating no significant multicollinearity. Sensitivity analysis using median-split categories for GTV and age yielded consistent directions of association with the main continuous-variable analysis. Reference groups and statistical methods are described in the Data analysis and statistical methods section.

In the univariate analysis, gender, tumor stage, and smoking status do not differentiate the mean ventilation for both the contralateral and ipsilateral lungs. In contrast, COPD and airway obstruction impose restrictive differences in mean ventilation for both the ipsilateral and contralateral lungs, while tumor location (central/peripheral) has a restrictive impact solely on the mean ventilation of the ipsilateral lung, without significant difference in the contralateral lung (P = 0.101). The mean ventilation for the ipsilateral lung consistently falls below that of the contralateral lung across all clinical factors, with 229 out of 274 individuals exhibiting this pattern. Spearman correlation analysis reveals a significant difference in mean ventilation for the ipsilateral lung associated with GTV volume (P = 0.015, r = -0.147), whereas no significant difference is observed in mean ventilation of the contralateral lung. Furthermore, neither age nor lung volume exhibit significant differences in mean ventilation for either lung.

In the multivariable analysis of the ipsilateral ventilation (**Table 2**), central tumor location (adjusted OR = 2.282, 95% CI: 1.320–3.945, P< 0.05), COPD (adjusted OR = 2.446, 95% CI: 1.386–4.317, P< 0.05), and airway obstruction (adjusted OR = 2.580, 95% CI: 1.185–5.618, P< 0.05) emerged as significant independent predictors of low ipsilateral ventilation. GTV volume (adjusted OR = 1.116 per 10 cm³, 95% CI: 0.825–1.495, P = 0.489) and age (adjusted OR = 1.072 per 10 years, 95% CI: 0.801–1.424, P = 0.637) did not show significant independent effects.​ Lung volume (excluded, adjusted OR = 1.000, P = 0.899) was also not significant. Similarly, in the contralateral lung analysis (**Table 3**), COPD (adjusted OR = 2.030, 95% CI: 1.191–3.459, P< 0.05) and airway obstruction (adjusted OR = 3.531, 95% CI: 1.606–7.761, P< 0.05) were significant independent predictors, while central tumor location showed a borderline significant association (adjusted OR = 1.666, 95% CI: 0.917–3.025, P = 0.094). Multicollinearity was assessed using VIF for all covariates in the multivariable models; all VIF values were below 5 (range: 1.011–1.12), indicating no significant collinearity.

The preceding paragraph introduced whether various clinical factors impact the ventilation of the ipsilateral and contralateral lungs of patients. However, prior to the decision on whether to implement functional-avoidance radiotherapy, it is imperative to comprehend not only the regional lung ventilation but also the heterogeneity of lung ventilation. Therefore, in **Table 4**, we employ a similar methodology to analyze the influences of the aforementioned clinical factors on the CoV of the ventilation. **Table 4** presents both univariate and multivariable results for CoV. In univariate analysis, significant differences in lung ventilation heterogeneity are observed with respect to tumor stage, tumor location, and airway obstruction. In multivariable analysis, airway obstruction emerged as the strongest independent predictor of elevated CoV (adjusted OR = 2.914, 95% CI: 1.315–6.454, P< 0.05). Central tumor location (adjusted OR = 1.614, 95% CI: 0.917–2.841, P = 0.097) and advanced stage (III–IV) (adjusted OR = 1.654, 95% CI: 0.942–2.903, P = 0.08) showed borderline significant associations with increased CoV, while COPD showed a trend toward lower CoV (adjusted OR = 0.604, 95% CI: 0.346–1.057, P = 0.077). For continuously coded clinical factors, GTV volume exhibited a positive trend with CoV in univariate analysis (r = 0.105, P = 0.084), but the association reversed direction and became non-significant in the multivariable model (adjusted OR = 0.895 per 10 cm³, 95% CI: 0.599–1.318, P = 0.565). Age and lung volume were not significantly associated with CoV in either univariate or multivariable analysis.

**Table 4:** The correlation between various clinical factors and the CoV of lung ventilation: univariate and multivariable analysis.

Characteristics	CoV	Univariate *P*	Adjusted OR (95% CI)	Multivariable *P*
Sex		0.831	1.256 (0.667-2.366)	0.48
Male	0.1534 ± 0.0737			
Female	0.1518 ± 0.0633			
Stage		< 0.05*	1.654 (0.942-2.903)	0.08
I-II	0.1244 ± 0.0525			
III-IV	0.1613 ± 0.0738			
Location		< 0.05*	1.614 (0.917-2.841)	0.097
Central	0.1721 ± 0.0802			
Peripheral	0.143 ± 0.0414			
COPD		0.268	0.604 (0.346-1.057)	0.077
Yes	0.1508 ± 0.0726			
No	0.1556 ± 0.0697			
Smoke		0.296	1.718 (0.883-3.342)	0.111
Yes	0.1551 ± 0.0714			
No	0.1499 ± 0.0711			
Airway obstruction		< 0.05*	2.914 (1.315-6.454)	< 0.05*
Yes	0.1712 ± 0.0575			
No	0.1496 ± 0.0731			
Age		0.577 (r = -0.034)	0.861 (0.644-1.149)	0.306
GTV volume		0.084 (r = 0.105)	0.895 (0.599-1.318)	0.565
Lung volume		0.238 (r = -0.072)		Excluded**

Data are presented as mean ± SD. High CoV was defined as ≥ median and coded as 1; low CoV (< median) was coded as 0. *P< 0.05. **Lung volume was excluded from the final multivariable model (adjusted OR = 0.999 per 1 cm³, 95% CI: 0.995–1.003, P = 0.565). GTV volume is reported per 10 cm³ increase; age is reported per 10-year increase. VIF values for all covariates in the multivariable model ranged from 1.011 to 1.12 (all< 5), indicating no significant multicollinearity. Sensitivity analysis using median-split categories for GTV and age yielded consistent directions of association with the main continuous-variable analysis. Reference groups and statistical methods are described in the Data analysis and statistical methods section.

### 3.2 Evaluation of dosimetric advantages for the functional-avoidance radiotherapy

Patients are categorized into four groups based on their lung ventilation heterogeneity (internal median splits), with eight patients selected from each group to create personalized functional-avoidance plans. Baseline clinical characteristics of these 32 patients were well-balanced across groups for sex, age, tumor location, COPD status, smoking history, GTV volume, and total lung volume (all P > 0.05; **Supplementary Table S1**). Notably, a significant gradient in airway obstruction was observed across the groups (P = 0.035), being most prevalent in Group 1 (50%) and absent in Groups 2 and 4. A similar trend was observed for disease stage (Stage III-IV: 100% in Group 1 vs. 37.5% in Group 4, P = 0.064), mirroring the clinical predictors identified in our multivariable analysis (**Table 4**).

The dose metrics for all four groups are presented in **Tables 5**–**8**. Statistical methods are employed to analyze the dosimetric differences of the target volumes and OARs between the functional-avoidance plans and anatomic plans within each group. **Table 5** outlines the dose metrics for Group 1 patients, encompassing dose metrics of target volumes, primary OARs, as well as entire lung and functional lung, from the functional-avoidance plans and anatomic treatment plans. No significant differences are observed in the CI and HI of the target volumes. However, the mean doses to the OARs excluding conventional and functional lungs in the functional-avoidance plans are all higher than those of the anatomic plans, albeit without reaching statistical significance. All conventional lung dose metrics (MLD, V5, V10, V20, and V30) and functional lung dose metrics (fMLD, fV10, fV20, and fV30) in the functional avoidance plans exhibit lower mean values compared to their counterparts in the anatomic plans. Notably, significant differences are detected in MLD and V20 of conventional lung dose metrics and fMLD, fV10, fV20, and fV30 of functional dose metrics between the two types of plans for this group.

**Table 5:** Dose metrics of target volumes and OARs from anatomic and functional-avoidance plans for Group 1 patients.

Variable	Anatomic IMRT	Functional IMRT	*P*
HI	0.84 ± 0.072	0.84 ± 0.063	0.969
CI	0.66 ± 0.139	0.63 ± 0.126	0.383
Heart V30 (%)	15.42 ± 11.25	16.02 ± 11.25	0.078
Heart V40 (%)	9.53 ± 7.18	9.86 ± 7.17	0.109
Heart mean (cGy)	1288.25 ± 815.52	1328.88 ± 824.89	0.148
Cord PRV, Dmax (cGy)	3839.6 ± 600.42	3924.24 ± 582.91	0.195
Esophagus mean (cGy)	1900.35 ± 615.73	1914.38 ± 632.41	0.547
Lung dose metrics			
MLD (cGy)	1062.04 ± 333.03	1033.37 ± 330.06	< 0.05*
V5 (%)	42.55 ± 8.86	42.04 ± 9.01	0.195
V10 (%)	25.86 ± 4.04	25.47 ± 3.74	0.109
V20 (%)	15.99 ± 4.21	15.16 ± 4.1	< 0.05*
V30 (%)	11.32 ± 2.43	11.07 ± 2.63	0.148
Functional lung dose metrics			
fMLD (cGy)	960.38 ± 421.29	931.25 ± 417.48	< 0.05*
fV5 (%)	38.49 ± 8.64	38.13 ± 8.54	0.250
fV10 (%)	23.38 ± 4.01	22.91 ± 4.12	< 0.05*
fV20 (%)	14.11 ± 4.35	13.43 ± 4.59	< 0.05*
fV30 (%)	10.65 ± 2.75	10.08 ± 2.78	< 0.05*

Data are presented as mean ± SD. *P< 0.05, compared with anatomic IMRT. HI, homogeneity index; CI, conformity index; Dmax, maximum dose; PRV, planning organ at risk volume.

**Table 6:** Dose metrics of target volumes and OARs from anatomic and functional-avoidance plans for Group 2 patients.

Variable	Anatomic IMRT	Functional IMRT	*P*
HI	0.86 ± 0.064	0.87 ± 0.06	0.641
CI	0.69 ± 0.155	0.68 ± 0.137	0.742
Heart V30 (%)	15.26 ± 8.01	16.14 ± 8.25	0.250
Heart V40 (%)	9.62 ± 8.02	9.72 ± 7.59	0.742
Heart mean (cGy)	1167.25 ± 611.75	1183.36 ± 624.6	0.148
Cord PRV, Dmax (cGy)	3758.88 ± 569.33	3842 ± 526.64	0.109
Esophagus mean (cGy)	1959.35 ± 581.26	1982.5 ± 585.04	0.148
Lung dose metrics			
MLD (cGy)	1150.79 ± 266.62	1131.12 ± 274.21	0.195
V5 (%)	45.05 ± 10.04	44.31 ± 10.49	0.250
V10 (%)	28.86 ± 5.47	28.36 ± 6.46	0.383
V20 (%)	17.36 ± 5.54	16.79 ± 5.91	0.078
V30 (%)	12.19 ± 3.17	11.96 ± 3.36	0.148
Functional lung dose metrics			
fMLD (cGy)	1030.38 ± 351.63	1000.1 ± 353.61	< 0.05*
fV5 (%)	39.745 ± 10.28	39.5 ± 10.26	0.383
fV10 (%)	24.63 ± 5.4	24.43 ± 5.49	0.461
fV20 (%)	15.74 ± 5.17	14.69 ± 5.04	< 0.05*
fV30 (%)	10.97 ± 3.32	10.62 ± 3.44	0.195

Data are presented as mean ± SD. *P< 0.05, compared with anatomic IMRT. HI, homogeneity index; CI, conformity index; Dmax, maximum dose; PRV, planning organ at risk volume.

**Table 7:** Dose metrics of target volumes and OARs from anatomic and functional-avoidance plans for Group 3 patients.

Variable	Anatomic IMRT	Functional IMRT	*P*
HI	0.84 ± 0.062	0.85 ± 0.057	0.852
CI	0.70 ± 0.127	0.69 ± 0.114	0.109
Heart V30 (%)	13.51 ± 5.9	14.55 ± 6.51	0.195
Heart V40 (%)	6.58 ± 4.15	6.56 ± 3.96	0.969
Heart mean (cGy)	1070.62 ± 563.52	1080.63 ± 576.36	0.383
Cord PRV, Dmax (cGy)	3792.16 ± 469.98	3868.32 ± 159.41	0.148
Esophagus mean (cGy)	1860.6 ± 690.94	1882.88 ± 681.61	0.250
Lung dose metrics			
MLD (cGy)	1023.04 ± 275.52	1010.63 ± 287.87	0.195
V5 (%)	40.49 ± 14.44	40.53 ± 14.92	0.969
V10 (%)	25.36 ± 8.93	25.01 ± 9.51	0.547
V20 (%)	15.73 ± 3.85	15.25 ± 3.69	< 0.05*
V30 (%)	11.13 ± 2.49	11.02 ± 2.56	0.148
Functional lung dose metrics			
fMLD (cGy)	959.75 ± 279.01	932.5 ± 277.45	< 0.05*
fV5 (%)	37.87 ± 13.8	37.62 ± 13.82	0.383
fV10 (%)	23.25 ± 8.92	23.18 ± 8.96	0.852
fV20 (%)	13.86 ± 3.82	13.36 ± 3.86	< 0.05*
fV30 (%)	10.59 ± 2.57	10.29 ± 2.71	0.461

Data are presented as mean ± SD. *P< 0.05, compared with anatomic IMRT. HI, homogeneity index; CI, conformity index; Dmax, maximum dose; PRV, planning organ at risk volume.

**Table 8:** Dose metrics of target volumes and OARs from anatomic and functional-avoidance plans for Group 4 patients.

Variable	Anatomic IMRT	Functional IMRT	*P*
HI	0.87 ± 0.073	0.87 ± 0.068	0.969
CI	0.73 ± 0.096	0.72 ± 0.097	0.195
Heart V30 (%)	12.84 ± 5.74	13.2 ± 6.26	0.313
Heart V40 (%)	6.39 ± 3.73	6.47 ± 3.77	0.461
Heart mean (cGy)	1033.87 ± 552.05	1045 ± 567.32	0.250
Cord PRV, Dmax (cGy)	3850.75 ± 490.9	3874.87 ± 460.09	0.547
Esophagus mean (cGy)	1603.5 ± 910	1609.2 ± 918.1	0.852
Lung dose metrics			
MLD (cGy)	1132.79 ± 338.2	1126.63 ± 341.46	0.250
V5 (%)	43.29 ± 11.79	42.31 ± 12.68	0.148
V10 (%)	28.49 ± 10.29	28.72 ± 10.72	0.641
V20 (%)	17.11 ± 3.89	17 ± 3.99	0.742
V30 (%)	11.92 ± 3.31	11.7 ± 3.64	0.461
Functional lung dose metrics			
fMLD (cGy)	1023.5 ± 200.86	1012.13 ± 201.63	0.195
fV5 (%)	38.75 ± 11.79	38.25 ± 11.96	0.148
fV10 (%)	24.38 ± 9.58	24.18 ± 9.52	0.641
fV20 (%)	14.86 ± 3.95	14.56 ± 4.24	0.250
fV30 (%)	10.47 ± 3.35	10.14 ± 3.38	0.313

Data are presented as mean ± SD. HI, homogeneity index; CI, conformity index; Dmax, maximum dose; PRV, planning organ at risk volume.

The dose metrics of the functional-avoidance plans and the anatomic plans for patients in Group 2 are listed in **Table 6**. Similar to Group 1, there are no significant differences in dose metrics of target volumes and OARs excluding conventional and functional lungs between the two types of plans for this group. The mean values of both conventional lung dose metrics and functional lung dose metrics in the functional-avoidance plans are consistently lower than those of the anatomic plans. However, among the five conventional lung dose metrics evaluated, no significant differences are observed between the two types of plans. In contrast, within the 5 functional lung dose metrics, fMLD and fV20 exhibit statistically significant differences.

The dose metrics of the functional-avoidance plans and the anatomic plans for patients in Group 3 are listed in **Table 7**. Consistent with the observations made in the previous two groups, no significant differences are noted in the dose metrics of target volumes and OARs excluding conventional and functional lungs between the two types of plans for Group 3 patients. Among the conventional lung dose metrics, there is a notable distinction in V20, while within the functional lung dose metrics, significant differences are observed in fMLD and fV20.

**Table 8** presents a comparison of dose metrics between the functional-avoidance plans and the anatomic plan for patients in Group 4. Unlike the previous three groups, the results show that there are no significant differences in all dose metrics of the target volumes, OARs between the two types of plans, including the conventional and functional lungs.

To rigorously evaluate the reliability of these observations, we performed FDR correction for multiple comparisons (**Supplementary Table S2**). After correction, the pattern of dosimetric benefit demonstrated a clear gradient across CoV quartiles. In Group 1 (highest CoV), key functional dose metrics, fMLD (Q = 0.040), fV10 (Q = 0.049), fV20 (Q = 0.049), and fV30 (Q = 0.049), remained statistically significant. In contrast, in Groups 2 and 3, even fMLD and fV20 did not reach significance after FDR correction (Q = 0.098), while in Group 4, no metrics reached significance after FDR correction.

Given the modest sample size of eight patients per group, a LOO sensitivity analysis was further conducted for the significant metrics in Group 1 to assess their robustness (**Supplementary Table S3**). This analysis revealed a spectrum of robustness: fMLD and fV20 demonstrated high robustness, remaining significant (P< 0.05) in all 8 of 8 LOO iterations with a consistent direction of change (functional< anatomic). Conversely, fV10 and fV30 were classified as “Borderline”, remaining significant in only 3 of 8 LOO iterations, likely reflecting limited statistical power rather than instability of the treatment effect. It should be emphasized that all above observations are based on dosimetric comparisons of replanned data without clinical follow-up for radiation pneumonitis, thus representing hypothesis-generating evidence of potential benefit rather than confirmed clinical patient-level outcomes.

### 3.3 Comparisons of individual patients

We select the most recently treated patients from each of the 8 patients undergoing functional-avoidance plan creations in Group 1 and Group 4, respectively, to compare the dose distributions and dose metrics between the functional-avoidance plan and anatomic plan. This comparison aims to illustrate the differences between functional-avoidance plans and anatomical plans in cases with stronger versus weaker ventilation heterogeneities. The patient from Group 1 has a CoV value of 0.177, ranking 44th out of 211 patients undergoing EBRT. The patient from Group 4 has a CoV value of 0.128, ranking 191st out of 211 patients undergoing EBRT.

**Figure 1** presents the dose distribution and dose-volume histogram (DVH) of key dose metrics for the two types of plans of the selected patient in Group 1. The upper left and upper right images depict the dose line distributions from the patient’s treatment plan and functional-avoidance plan on the same CT image. The red structures in the images represent the functional lung, while the green structure represents the PTV (for this patient, the PGTV is not prescribed a dose), with the prescription dose for PTV set to 60 Gy. The prescription dose coverage for PTV remains at around 95.5% for both plans. The ventilation capacity is relatively better on the left and upper left sides of the PTV, while it is poorer on the lower left side. However, in the treatment plan, the 20 Gy dose line covers part of the functional lung on the upper left side. Therefore, in the functional-avoidance plan, this area is specially protected, along with the highly functional area below the PTV. Additionally, the beam arrangement for the patient’s functional-avoidance plan is consistent with that of the treatment plan. The lower part of **Figure 1** details the differences in key dose metrics between the two types of plans. The values of the functional dose metrics fMLD, fV5, fV10, fV20, and fV30 in the functional-avoidance plan are 10.7%, 1.16%, 1.53%, 31.8%, and 27.8% smaller than the corresponding values of the anatomic plan, respectively. The mean dose to the Cord PRV increases slightly, but the maximum dose remains similar, and the doses to other critical OARs increase marginally.

**Figure 1:**
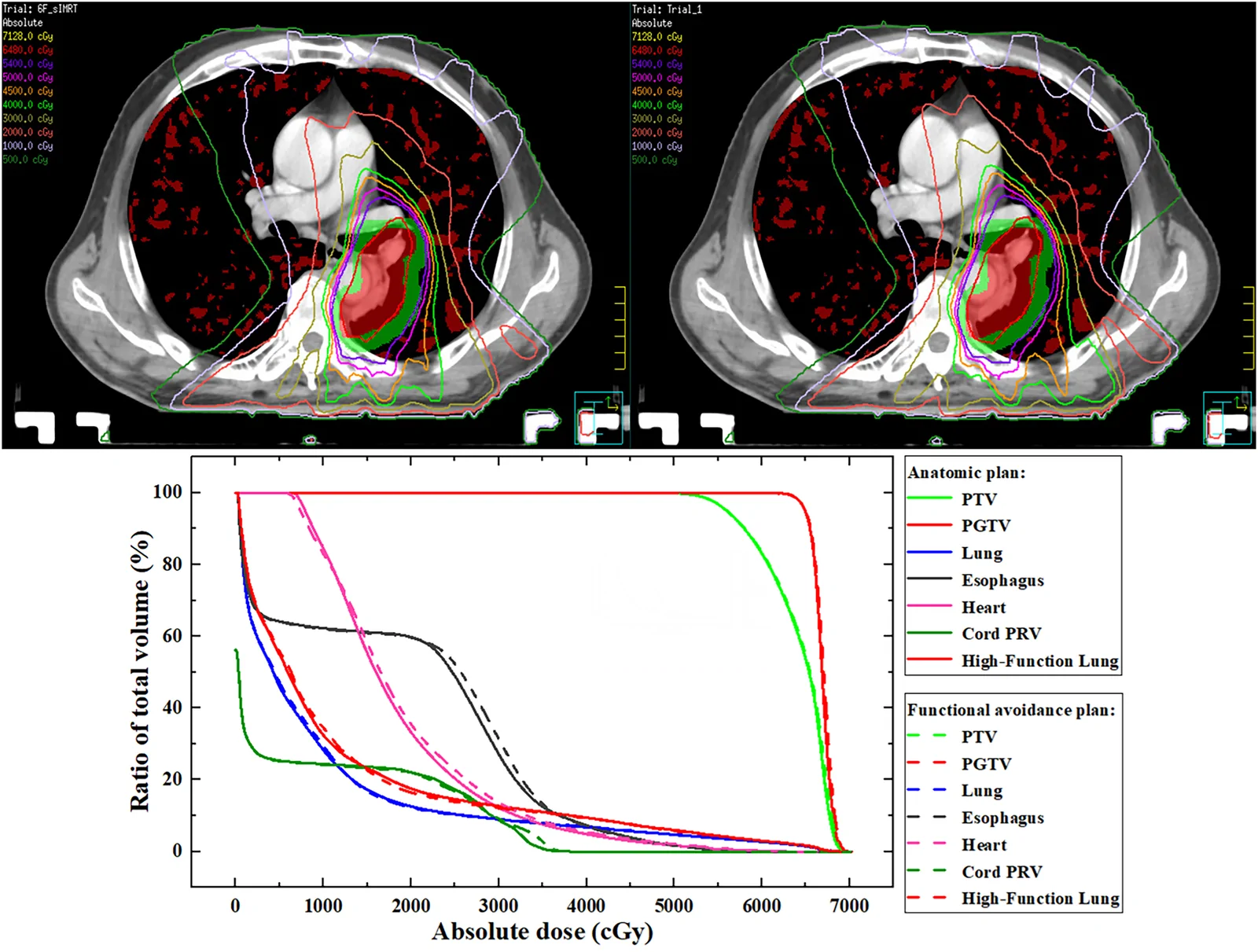
Dose distributions and dose-volume histogram (DVH) curves for the anatomic and functional-avoidance plans (Group 1 patient).​ Upper row: axial dose lines overlaid on CT. Line colors correspond to absolute doses (cGy) shown in the top-left legend. Green contours indicate the planning target volume (PTV), and red contours indicate the high-function lung (top 20% ventilation). Lower row: DVH comparisons for PTV, lung, esophagus, heart, spinal cord planning organ at risk volume (Cord PRV), and high-function lung. Solid lines represent the anatomic plan; dashed lines represent the functional-avoidance plan. These images illustrate the dosimetric differences between plan types; clinical validation with radiation pneumonitis endpoints is pending.

Similar to **Figures 1**, **2** presents the dose line distributions and DVH of key dose metrics for the two types of plans of the selected patient in Group 4. The upper left and upper right images depict the dose line distributions from the patient’s treatment plan and functional-avoidance plan on the same CT image. The red structures within the lung volume represent the functional lung, the green structure represents the PTV and the red structure within the PTV is the PGTV. The prescription doses for PGTV and PTV are 64.8 Gy and 54 Gy, respectively. Both plans achieve prescription dose coverage of approximately 96.3% for PGTV and 95% for PTV. Due to the uniform distribution of highly functional areas around PTV, there is limited room for dose optimization in these highly functional areas. Consequently, there is little difference in dose distributions between the two plans, as evident in the lower part of **Figure 2**. The dose to the functional lung decreases slightly, while the doses to the heart and esophagus increase marginally. These individual case demonstrations highlight the spatial rationale for functional avoidance, though clinical validation with radiation pneumonitis endpoints remains pending.

**Figure 2:**
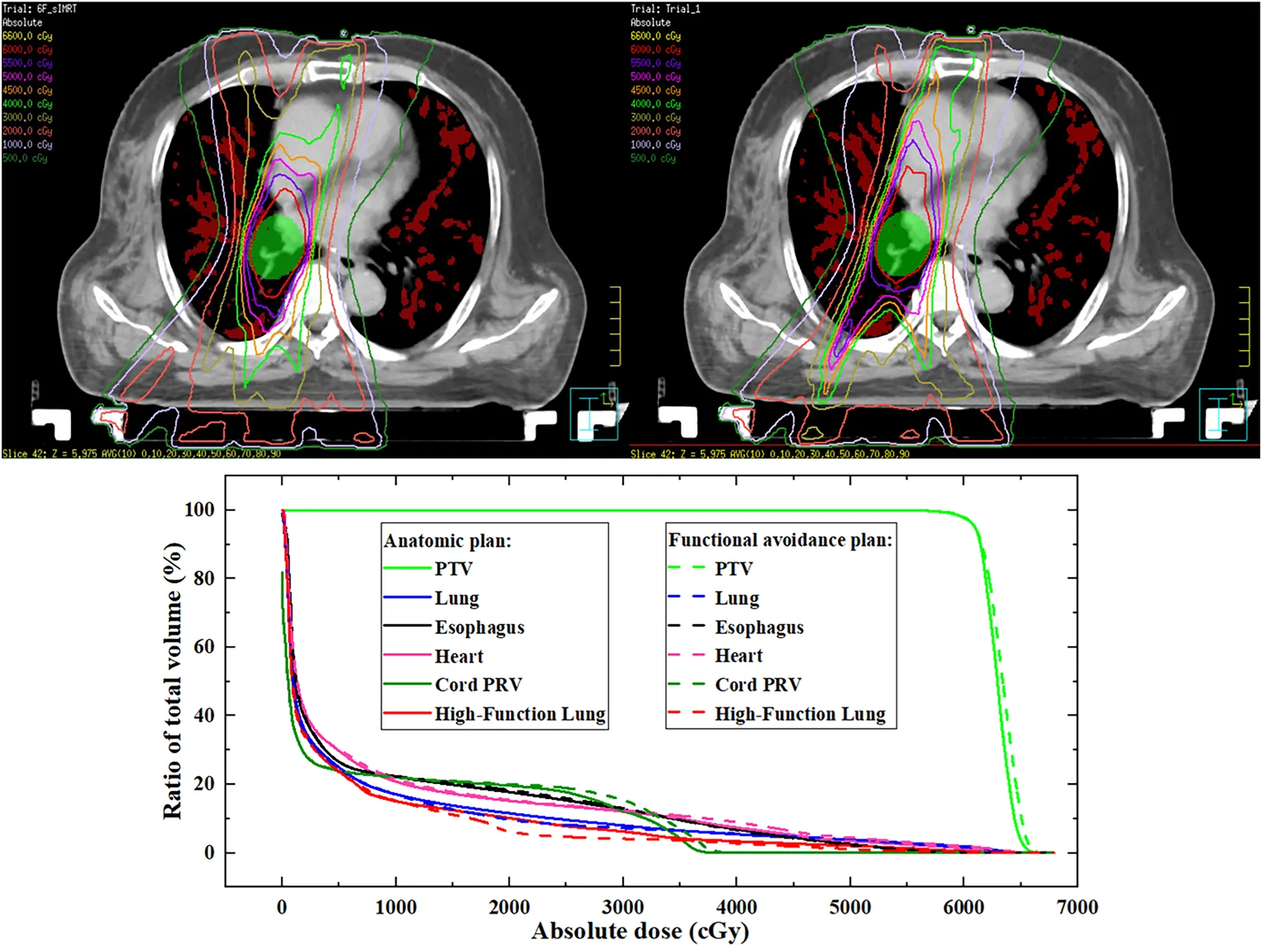
Dose distributions and dose-volume histogram (DVH) curves for the anatomic and functional-avoidance plans (Group 4 patient).​ Upper row: axial dose lines overlaid on CT. Line colors correspond to absolute doses (cGy) shown in the top-left legend. Green contours indicate the planning target volume (PTV), and red contours indicate the high-function lung (top 20% ventilation). Lower row: DVH comparisons for PTV, lung, esophagus, heart, spinal cord planning organ at risk volume (Cord PRV), and high-function lung. Solid lines represent the anatomic plan; dashed lines represent the functional-avoidance plan. These images illustrate the dosimetric differences between plan types; clinical validation with radiation pneumonitis endpoints is pending.

## 4 Discussion

This research endeavor involves an in-depth analysis of 274 lung cancer patients, examining the correlation between various key clinical factors and the heterogeneity of patients’ regional lung ventilation function. Furthermore, we compare the dosimetric differences between the functional-avoidance plans and anatomic plans, aiming to ascertain the potential dosimetric benefits of functional-avoidance radiotherapy for patients with certain clinical factors. To our knowledge, this is the first study to incorporate multiple clinical factors into both univariate and multivariable analyses of patients’ lung ventilation function and its heterogeneity, and to identify patients under which clinical factors are more likely to benefit from functional-avoidance radiotherapy.

### 4.1 Clinical factors associated with regional lung ventilation

Regarding the impacts of various factors on regional lung ventilation (**Tables 2**, **3**), univariate analysis revealed no significant differences in mean ventilation according to gender, tumor stage, or smoking status for either lung. Notably, smokers showed higher mean ventilation in both lungs compared to non-smokers, which may partly reflect the fact that most female patients in our cohort were non-smokers and had slightly lower mean ventilation than male patients. Patients with stage I–II tumors demonstrated lower mean ventilation than those with stage III–IV tumors, a finding that may be attributed to selection bias at our institution, where stage I–II patients with better baseline lung function are more likely to undergo surgery rather than radiotherapy, leaving a cohort of stage I–II patients with relatively compromised pulmonary function.

In contrast, tumor location, COPD, airway obstruction, and GTV volume were significantly associated with ventilation in at least one lung in univariate analysis. COPD inhibits respiration and thereby reduces lung ventilation. The impacts of tumor location, airway obstruction, and GTV volume on ventilation are likely interrelated: larger, centrally located tumors are more likely to cause airway obstruction, which in turn compromises regional ventilation (the mean GTV volume in patients with airway obstruction was 1.33 times that of the overall cohort, and 39 of 43 patients with airway obstruction had central tumors). Furthermore, across all clinical factors, mean ipsilateral ventilation was consistently lower than contralateral ventilation (229 of 274 patients, 83.6%), underscoring the direct impact of the tumor and any associated obstruction on ipsilateral lung function.

To identify which of these factors independently predict regional ventilation, we performed multivariable logistic regression. For ipsilateral ventilation (**Table 2**), central tumor location (adjusted OR = 2.282, 95% CI: 1.320–3.945, P< 0.05), COPD (adjusted OR = 2.446, 95% CI: 1.386–4.317, P< 0.05), and airway obstruction (adjusted OR = 2.580, 95% CI: 1.185–5.618, P< 0.05) remained significant independent predictors. Stage and smoking status did not show significant associations with regional ventilation in either univariate or multivariable analyses. For contralateral ventilation (**Table 3**), only COPD (adjusted OR = 2.030, 95% CI: 1.191–3.459, P< 0.05) and airway obstruction (adjusted OR = 3.531, 95% CI: 1.606–7.761, P< 0.05) were significant independent predictors, while central tumor location showed a borderline significant association (adjusted OR = 1.666, 95% CI: 0.917–3.025, P = 0.094). The attenuation of the effect of tumor location on contralateral ventilation in the multivariable model is consistent with a localized rather than systemic mechanism: central tumors primarily affect the lung on the side of the obstruction, with limited contralateral impact.

### 4.2 Mechanisms underlying ventilation heterogeneity

Among the effects of various clinical factors on ventilation heterogeneity (**Table 4**), the multivariable analysis revealed that airway obstruction was the strongest independent predictor of elevated CoV (adjusted OR = 2.914, P< 0.05). Central tumor location (OR = 1.614, P = 0.097) and advanced stage (III–IV) (OR = 1.654, P = 0.08) showed borderline significant associations, while COPD showed a trend toward lower CoV (OR = 0.604, P = 0.077). The loss of statistical significance for central location in the multivariable model may reflect shared variance with airway obstruction, as central tumors more frequently cause bronchial obstruction; the consistent direction of effect across both variables, however, supports the biological link between focal airway compromise and ventilation heterogeneity.

The biological chain is as follows: central tumors, arising from segmental bronchi or larger airways, may cause partial or complete obstruction through direct luminal invasion, extrinsic compression, or mucus plugging. When a bronchus is partially obstructed, distal lung tissue becomes hypo-ventilated while remaining patent; when obstruction becomes complete, the trapped air distal to the blockage may be gradually absorbed, leading to obstructive (absorption) atelectasis and volume loss in the affected region. Concurrently, post-obstructive pneumonia may develop due to impaired mucociliary clearance and secondary infection. These processes create a sharp, localized defect in ventilation within the obstructed territory, while the non-obstructed lung regions maintain relatively normal ventilation. This focal, regional pattern of ventilation loss—unlike the diffuse involvement characteristic of COPD, produces a marked spatial gradient in ventilation distribution, thereby elevating the CoV across the six lung regions.

In contrast to the focal mechanism of tumor-related obstruction, COPD appears to exert a different effect on CoV.​ COPD is a heterogeneous disease, and severe emphysema can indeed lead to heterogeneous lung ventilation patterns. In our multivariable analysis, COPD showed a trend toward lower CoV (adjusted OR = 0.604, 95% CI: 0.346–1.057, P = 0.077), which did not reach formal statistical significance but suggests a potential pattern of reduced rather than increased ventilation heterogeneity. This may be explained by the diffuse nature of COPD: unlike a focal central obstruction which creates a sharp contrast between a hypo-ventilated obstructed region and normally ventilated parenchyma, COPD is a predominantly diffuse airway and parenchymal disease. The resulting ventilation reduction tends to be broadly distributed rather than sharply localized, limiting the development of strong spatial ventilation gradients. However, in patients with advanced, heterogeneous emphysema, COPD could potentially increase CoV, a nuance our current cohort may not fully capture.

GTV volume showed a positive trend with CoV in univariate analysis (r = 0.105, P = 0.084), whereas the association reversed direction and became non-significant in the multivariable model (adjusted OR = 0.895 per 10 cm³, 95% CI: 0.599–1.318, P = 0.565). This suppression pattern suggests that the apparent positive association in univariate analysis may have been driven by confounding with tumor location and airway obstruction, factors that independently predict higher CoV and are correlated with larger tumor size. After adjusting for these variables, GTV volume itself showed minimal independent association with ventilation heterogeneity.

Our study concurs with previous research (17) in identifying a significant difference in lung ventilation heterogeneity associated with tumor stage. This association may stem from the larger tumor volumes (mean GTV volume: 63.69 cm³ for stage III–IV versus 48.47 cm³ for stage I–II) and higher proportion of airway obstruction (39/213 for stage III–IV versus 4/61 for stage I–II) among advanced-stage patients. However, as noted above, GTV volume itself did not retain an independent association with CoV after adjusting for tumor location and airway obstruction, suggesting that tumor size contributes to ventilation heterogeneity primarily through its correlation with these factors rather than as an independent driver.​ In summary, tumor stage, tumor location, and airway obstruction are strongly correlated with patients’ lung ventilation heterogeneity. Patients with airway obstruction and central tumors of stage III–IV tend to have more pronounced ventilation heterogeneity.

### 4.3 Dosimetric benefits of functional-avoidance radiotherapy

In comparing the functional-avoidance plans with the anatomic treatment plans for patients exhibiting variations in ventilation heterogeneity, we exclude those undergoing SBRT due to the substantial differences in planning strategies, dose requirements, and the relatively low prevalence of SBRT compared to conventional EBRT. Our results demonstrate that functional-avoidance radiotherapy effectively reduces the dose to functional lung without compromising target coverage, albeit to a degree that is strongly dependent on baseline ventilation heterogeneity.​ Group 1 patients experience the most pronounced decreases in various functional lung dose metrics, while Group 4 shows the least significant reductions. Specifically, 4 out of 5 functional lung dose metrics (fMLD, fV10, fV20, fV30) in Group 1, 2 in both Group 2 (fMLD, fV20) and Group 3 (fMLD, fV20), and none in Group 4 exhibit significant differences between the two types of plans. This consistent dose-response gradient, from 4 significant metrics in Group 1 to 0 in Group 4, supports a biological and dosimetric relationship rather than random variation. Crucially, this pattern remained evident after FDR correction: in Group 1 (highest CoV), key metrics including fMLD, fV10, fV20, and fV30 remained significant (Q = 0.040–0.049), whereas significance was attenuated or lost in groups with lower heterogeneity.​ Furthermore, LOO sensitivity analysis confirmed fMLD and fV20 as robust endpoints in Group 1, whereas fV10 and fV30 were more sensitive to sample composition, suggesting that future studies should prioritize these stable metrics.​ Nevertheless, these findings remain exploratory and require prospective validation.

The significant reductions in fMLD and fV20 observed in Group 1 and the nominal reductions seen in Groups 2 and 3 likely reflect both the avoidance strategy employed by planners and the underlying physiological distribution of lung function.​ Planners often assigned higher avoidance weights to these metrics, which represent both the overall mean dose and the dose to highly functional subvolumes. Importantly, the optimization of beam arrangements corroborates this physiological rationale: beam angles differed from the anatomic plans in 5/8, 2/8, 3/8, and 0/8 patients across Groups 1 to 4, respectively. This gradient reflects clinical reality and does not constitute a confounding bias in our paired design; patients with high ventilation heterogeneity (Group 1) present with distinct, localized regions of functional lung, providing planners with the spatial opportunity to employ more aggressive optimization strategies (e.g., beam angle modification) to spare critical areas. Conversely, in patients with homogeneous ventilation (Group 4), there is limited room to alter beam paths without compromising target coverage. Thus, the observed dosimetric advantages are intrinsically linked to the spatial heterogeneity of lung function, which provides the necessary window for functional avoidance.

Furthermore, while functional-avoidance radiotherapy significantly reduced doses to functional lung and total lung, it induced only minor, non-significant increases in doses to other organs-at-risk (OARs), such as the heart.​ This trade-off is clinically acceptable given the priority of preserving pulmonary function. It is also worth noting that patients with target volumes extending over a longer superior-inferior range may derive even greater benefit from functional avoidance, as they typically have more lung tissue at risk; however, this specific aspect was not investigated in the current study.

In conclusion, while our findings highlight a heightened dosimetric benefit of functional-avoidance radiotherapy in patients with specific clinical factors (e.g., airway obstruction, advanced stage, central location), it is important to note that the CoV cut-offs used for stratification were derived from internal median splits within this cohort and do not represent universally validated clinical thresholds.​ Therefore, these factors should not yet be regarded as rigid clinical criteria.​ Instead, they serve as valuable references for patient selection in future prospective trials, suggesting that patients with these characteristics are more likely to achieve meaningful dose reductions to functional lung compared to those without them. It must be emphasized that, as a plan-comparison study without radiation pneumonitis follow-up, the observed advantages are strictly dosimetric and hypothesis-generating, rather than evidence of direct clinical patient-level benefit.

### 4.4 Limitations

This study has several limitations across four major domains.

First, selection bias. This is a single-center retrospective study, and only 32 of 274 patients underwent re-planning, with 8 patients per quartile. Our analysis employed a paired design, with each patient serving as their own control, which eliminates inter-patient variability and increases statistical power relative to independent-sample designs. For paired comparisons with small samples, the Wilcoxon signed-rank test provides exact P-values for n ≤ 25, and n=8 is well within the valid range for exact testing. To address the issue of multiple comparisons, FDR correction was applied as described in the Methods section.​ Furthermore, LOO sensitivity analyses were conducted for significant functional metrics in Group 1 to assess robustness against sample composition.​ Nonetheless, the modest sample size limits statistical power for detecting subtle dose differences, and the results should be regarded as exploratory hypothesis-generating rather than definitive evidence. The results require prospective validation in larger, multi-center cohorts. In addition, we excluded SBRT patients from the dosimetric comparison due to substantial differences in planning strategies and dose requirements, which may limit the generalizability of our findings to the SBRT population. Therefore, the dosimetric findings of this study apply only to conventional fractionated IMRT and should not be extrapolated to SBRT populations, and these results should be interpreted as hypothesis-generating evidence rather than definitive clinical proof.

Second, measurement bias. 4D-CT ventilation calculation involves deformable image registration (38), ventilation computation methods (39) and normalization procedures (40), which could carry inherent uncertainties. Deformable image registration is a major source of measurement uncertainty. The estimated deformation field, and therefore the HU-based ventilation map, depends on image quality, quantum noise, artifact levels, respiratory motion amplitude, phase sorting, segmentation consistency, and algorithm choices such as grid spacing, similarity metric, and the regularization weight λ. Different DIR implementations or parameter settings can yield systematically different voxel correspondences and thus different regional ventilation values, altered six-zone CoV, and shifted spatial definition of the top 20% functional lung used for avoidance planning (13, 38). The present study adopted the Plastimatch cubic B-spline configuration described above (13, 33), but no gold-standard deformation field was available for this cohort; therefore DIR-related variability should be considered when interpreting CoV groupings and dosimetric comparisons. Furthermore, the CoV quartiles used for stratification were derived from internal median splits within this cohort and represent hypothesis-generating cut-offs; external validation in independent cohorts is required to establish definitive clinical thresholds. Free-breathing acquisition, anatomic discontinuities, and sliding motion at the pleural boundary may further limit registration fidelity despite manual quality control.

Regarding the functional lung definition, we defined the functional lung as the top 20% ventilation region for avoidance. This threshold was selected because it sits within the most commonly adopted range (10%–30%) in the field of functional avoidance radiotherapy (20–28). Prior literature has established that, across a broad range of thresholds, the choice of ventilation threshold does not drastically alter predictive outcomes (21). Given this demonstrated stability, we selected the top 20% as a representative cutoff for functional lung definition in this study.​ However, a systematic sensitivity analysis using alternative thresholds was beyond the scope of the current work. A comprehensive comparison of multiple thresholds remains a distinct research question warranting dedicated future investigation. Additionally, the significant differences observed in fMLD and fV20 across Groups 1, 2, and 3 may be partly attributed to the avoidance strategy employed by planners, who likely assigned a higher avoidance weight to these metrics. Different avoidance strategies may lead to varying outcomes, and our findings should be interpreted with this in mind.

Third, confounding bias. Unmeasured covariates such as detailed smoking pack-years, prior thoracic interventions, and non-COPD pulmonary comorbidities may influence ventilation heterogeneity. In the multivariable analysis, GTV volume showed a positive trend with CoV in univariate analysis but the association reversed direction and became non-significant in the multivariable model, suggesting that the apparent positive association may have been driven by confounding with tumor location and airway obstruction. We adjusted for these factors in the multivariable model, but residual confounding from unmeasured variables cannot be ruled out.

Fourth, translation bias. The absence of follow-up radiation pneumonitis data limits our ability to directly verify clinical protective effects; thus, the dosimetric benefits identified in this retrospective study represent plan-comparison evidence only and require prospective clinical validation with long-term patient outcomes before direct clinical implementation. Nevertheless, prior studies have established that the dose to highly functional lung regions directly influences the risk of radiation pneumonitis (22), and others have demonstrated clinical benefits of functional-avoidance radiotherapy, including improved quality of life (31). These findings provide clinical context to support the translational relevance of our dosimetric results. A quantitative CoV threshold for clinical screening could be valuable for future prospective studies. We acknowledge that the CoV quartiles used in this study were derived from internal median splits and represent hypothesis-generating cut-offs. External validation in independent cohorts is required to establish definitive clinical thresholds for patient selection.

## 5 Conclusions

This study investigated the correlation between clinical factors and regional lung ventilation as well as ventilation heterogeneity in 274 lung cancer patients. In multivariable analyses of ipsilateral and contralateral mean ventilation and CoV, airway obstruction consistently emerged as the strongest independent predictor across all three outcomes. Central tumor location and COPD were also significant predictors of impaired regional ventilation, while GTV volume and lung volume showed minimal independent effects after adjusting for confounders. When comparing functional-avoidance plans with anatomic plans across patients with varying degrees of ventilation heterogeneity, patients with stronger heterogeneity were more likely to derive dosimetric benefit from functional-avoidance radiotherapy. Based on these findings, lung cancer patients with central tumors, airway obstruction, and advanced stage, who exhibit the strongest ventilation heterogeneity, may represent appropriate candidates for functional-avoidance radiotherapy in future prospective trials. However, since this replanning study lacked clinical follow-up for radiation pneumonitis, these findings reflect dosimetric rather than confirmed clinical patient-level benefit and require prospective validation.​ Notably, the CoV cut-offs employed were derived from internal median splits within this cohort and require external validation. This study provides a dosimetric basis for hypothesis-generating patient selection and offers a reference to guide clinical decision-making regarding the adoption of functional-avoidance radiotherapy.

## Data Availability

The raw data supporting the conclusions of this article will be made available by the authors, without undue reservation.

## Supplementary material

SUPPLEMENTARY TABLE S1 Baseline balance data for all four CoV subgroups.

SUPPLEMENTARY TABLE S2 False Discovery Rate (FDR) correction results distinguishing robust findings (Group 1) from nominal trends (Groups 2–4).

SUPPLEMENTARY TABLE S3 Leave-one-out (LOO) sensitivity analysis results for dosimetric endpoints.
