# Supplementary material for: Relevance between patients’ clinical factors and 4D-CT lung ventilation heterogeneity for evaluating the adoption of functional avoidance thoracic radiotherapy with a large patient database
Source: Front Oncol. 2026 Oct 7;16:1955295. doi: 10.3389/fonc.2026.1955295 (PMC13682974; doi:10.3389/fonc.2026.1955295)
Supplement: Supplementary file 1 [file Table1.docx]

**Table S1. Baseline clinical characteristics of the 32 replanned patients across the four CoV-based groups (8 per group).**

| Variable | Group 1 | Group 2 | Group 3 | Group 4 | Over all P |
| --- | --- | --- | --- | --- | --- |
| Sex, male n (%) | 7 (87.5) | 8 (100) | 7 (87.5) | 5 (62.5) | 0.342 |
| Stage, III-IV n (%) | 8 (100) | 6 (75) | 5 (62.5) | 3 (37.5) | 0.064 |
| Tumor location, central n (%) | 7 (87.5) | 4 (50) | 3 (37.5) | 3 (37.5) | 0.171 |
| COPD, yes n (%) | 6 (75) | 3 (37.5) | 4 (50) | 3 (37.5) | 0.424 |
| Smoking, yes n (%) | 7 (87.5) | 5 (62.5) | 4 (50) | 4 (50) | 0.397 |
| Airway obstruction, yes n (%) | 4 (50) | 0 (0) | 1 (12.5) | 0 (0) | 0.035* |
| Age, median (range) | 68 (51-80) | 66 (62-71) | 73 (50-87) | 73 (43-83) | 0.409 |
| GTV volume, median (range) cm³ | 34.12 (9.71-70.81) | 18.77 (4.33-314.18) | 41.05 (9.71-262.33) | 14.17 (2.62-62.9) | 0.504 |
| Total lung volume, median (range) cm³ | 3357.4 (2256.8-4222.6) | 2843.1 (2078.7-4011.4) | 3224.8 (2657.5-4691.0) | 3397.0 (2440.1-4237.4) | 0.603 |

Note: Categorical variables were compared with the Fisher–Freeman–Halton exact test, and continuous variables with the Kruskal–Wallis test; each row reports one overall P-value testing for differences across the four CoV subgroups. Airway obstruction differed significantly across groups (P = 0.035), consistent with its role as a predictor of high CoV. The remaining key confounders (age, sex, disease stage, GTV volume, smoking, COPD, total lung volume) were well balanced at baseline (all P > 0.05).

Table S2. False Discovery Rate (FDR) correction for paired dosimetric comparisons across the four CoV-based groups (Group 1 = highest to Group 4 = lowest CoV quartile).

| Metric | Group 1 Raw P (FDR Q) | Group 2 Raw P (FDR Q) | Group 3 Raw P (FDR Q) | Group 4 Raw P (FDR Q) |
| --- | --- | --- | --- | --- |
| Conventional lung metric |  |  |  |  |
| MLD | 0.023* (0.098) | 0.195 (0.313) | 0.195 (0.325) | 0.250 (0.625) |
| V5 | 0.195 (0.195) | 0.250 (0.313) | 0.969 (0.969) | 0.148 (0.625) |
| V10 | 0.109 (0.182) | 0.383 (0.383) | 0.547 (0.684) | 0.641 (0.742) |
| V20 | 0.039* (0.098) | 0.078 (0.313) | 0.039* (0.195) | 0.742 (0.742) |
| V30 | 0.148 (0.185) | 0.148 (0.313) | 0.148 (0.325) | 0.461 (0.742) |
| Functional lung metric |  |  |  |  |
| fMLD | 0.008* (0.040**) | 0.039* (0.098) | 0.023* (0.098) | 0.195 (0.391) |
| fV5 | 0.250 (0.250) | 0.383 (0.461) | 0.383 (0.576) | 0.148 (0.391) |
| fV10 | 0.039* (0.049**) | 0.461 (0.461) | 0.852 (0.852) | 0.641 (0.641) |
| fV20 | 0.023* (0.049**) | 0.039* (0.098) | 0.039* (0.098) | 0.250 (0.391) |
| fV30 | 0.039* (0.049**) | 0.195 (0.325) | 0.461 (0.576) | 0.313 (0.391) |

**Note:** Within each CoV group, FDR correction was performed separately within two pre-specified families using the Benjamini–Hochberg (BH) procedure: conventional whole-lung metrics (MLD, V5, V10, V20, V30; m=5) and functional-lung metrics (fMLD, fV5, fV10, fV20, fV30; m=5). This avoids cross-family penalty while controlling the false discovery rate within each family. Raw P values were obtained from the two-sided exact paired Wilcoxon signed-rank test comparing anatomic and functional-avoidance plans. Raw P < 0.05 is marked with *; FDR-adjusted Q < 0.05 is marked with **. FDR Q < 0.05 was considered statistically significant after correction.

Abbreviations: MLD, mean lung dose; fMLD, functional MLD; Vx, percentage of lung volume receiving ≥ x Gy; fVx, functional lung Vx.

**Table S3.** Leave-one-out (LOO) sensitivity analysis for functional dose metrics remaining significant after FDR correction in Group 1 (highest CoV quartile).

| Metric | Full cohort P (n=8) | | LOO1 | LOO2 | LOO3 | LOO4 | LOO5 | LOO6 | LOO7 | LOO8 | n significance/8 | Direction consistent | Conclusion |
| --- | --- | --- | --- | --- | --- | --- | --- | --- | --- | --- | --- | --- | --- |
| fMLD | | 0.008 | 0.016 | 0.016 | 0.016 | 0.016 | 0.016 | 0.016 | 0.016 | 0.016 | 8/8 | Yes | Robust |
| fV10 | | 0.039 | 0.047 | 0.047 | 0.016 | 0.078 | 0.078 | 0.078 | 0.078 | 0.078 | 3/8 | Yes | Borderline |
| fV20 | | 0.023 | 0.031 | 0.016 | 0.047 | 0.047 | 0.047 | 0.047 | 0.047 | 0.047 | 8/8 | Yes | Robust |
| fV30 | | 0.039 | 0.047 | 0.047 | 0.016 | 0.078 | 0.078 | 0.078 | 0.078 | 0.078 | 3/8 | Yes | Borderline |

Note: For each metric separately, one patient is removed according to the rank of the absolute paired difference for that metric (LOO1 = rank 1, LOO2 = rank 2, …, LOO8 = rank 8), and the exact two-sided Wilcoxon signed-rank test is re-run on the remaining 7 patients. P values and direction of change (functional < anatomic) are recorded. A metric was classified as Robust if it remained significant (P < 0.05) in ≥6 of 8 LOO runs with a consistent direction of change, and as Borderline otherwise. Metrics not significant after FDR correction (Groups 2–4) were not subjected to LOO.
